# Clinical Resolution of Osmotic Demyelination Syndrome following Overcorrection of Severe Hyponatremia

**DOI:** 10.1155/2019/1757656

**Published:** 2019-03-20

**Authors:** Ruhin Yuridullah, Vinod Kumar, Sushant Nanavati, Monisha Singhal, Chandra Chandran

**Affiliations:** Saint Joseph's University Medical Center, USA

## Abstract

Osmotic Demyelination Syndrome (ODS) occurs after rapid overcorrection of severe chronic hyponatremia usually in those with a predisposition such as chronic alcoholism, malnutrition, or liver disease. Rarely, do patients make a full recovery. We report a case of ODS secondary to overcorrection of severe hyponatremia with pathognomonic clinical and radiologic signs making a complete neurological recovery. A detailed course of events, review of literature, and optimal and aggressive management strategies are discussed. There is some controversy in the literature regarding the prognosis of these patients. Our aim here is to show that, with aggressive therapy and long-term care, recovery is possible in these patients.

## 1. Introduction

Osmotic Demyelination Syndrome (ODS) also known as central pontine myelinolysis (CPM) was first described by Adams et al. in 1958 in alcoholics and malnourished who then developed spastic quadriplegia, pseudobulbar palsy, and varying degrees of encephalopathy or coma from acute, noninflammatory demyelination of the pons [1]. The ODS primarily occurs with overly rapid correction of severe hyponatremia (serum sodium <120 mEq/L) that has been present for more than two to three days [2–7]. The majority of ODS cases occur when initial sodium concentrations are ≤105 mEq/L [4, 5, 7, 8]. Sterns et al. showed that correction by >12 mEq/L in 24 hours or >18 mEq/L in 48 hours was associated with posttherapeutic neurologic complications when initial sodium concentration was ≤105 mEq/L [5].

Several factors appear to increase susceptibility to ODS including alcoholism, malnutrition, liver disease, and hypokalemia and the duration of hyponatremia [4, 5, 8–12]. Studies in animals have shown that brain damage does not occur when hyponatremia of <1 day duration is rapidly corrected. However, the same treatment results in fatal demyelination if hyponatremia has persisted for >2 days [9, 13–15].

The clinical manifestations of ODS are typically delayed for 2-6 days after rapid overcorrection of severe hyponatremia [5, 7, 8, 16]. The symptoms, often irreversible or partially reversible include dysarthria, dysphagia, paraparesis or quadriparesis, movement disorders, confusion, disorientation, obtundation, and coma [3, 5–7, 17]. Severely affected patients may become “locked in”; they are awake but unable to move or communicate.

Treatment often involves relowering of the sodium concentration and aggressive supportive management usually in the intensive care setting [16, 18, 19]. Death is common usually secondary to associated complications (ventilator dependency, pneumonia, venous thrombosis, pulmonary embolism, and muscle wasting) with recovery, often partial, following several months [20, 21].

## 2. Case Presentation

A 54-year-old male with chronic alcoholism presented with altered mental status. He had no other past medical history apart from multiple presentations to emergency services for alcohol intoxication/withdrawal symptoms. He was not on any medications. On presentation, his vital signs were within normal limits. His Glasgow Coma Scale (GCS) was 15. He was alert, awake, but disoriented to time. Physical examination was unrevealing. CT scan of the brain was negative for any acute pathology. Laboratory evaluation revealed serum alcohol level of <10 mg/dL and urine drug toxicology was negative.

His complete metabolic panel revealed a serum sodium concentration of 102 mEq/L, potassium 2.4 mEq/L, chloride 54 mEq/L, bicarbonate 38 mEq/L, blood urea nitrogen 8 mg/dL, creatinine 0.62 mg/dL, magnesium 2.2 mg/dL, phosphorous 2.3 mg/dL, albumin 3.6 g/dL, alkaline phosphatase 116 U/L, aspartate aminotransferase 117 U/L, alanine aminotransferase 122 U/L, and bilirubin 0.9 mg/dL. His plasma osmolality was 212 mOsm/kg.

In the emergency department, he received two 1-liter boluses of 0.9% saline intravenously. His serum potassium was replete with potassium chloride 40 mEq intravenously and 80 mEq orally. He was then started on intravenous 0.9% saline infusion at a rate of 100 mL/hr. Serum sodium concentrations and other electrolytes were monitored periodically (Table 1). His serum sodium concentration was 106 mEq/L by 8 hours but increased to 112 mEq/L by 16 hours, at which point 0.9% saline infusion rate was decreased to 60 mL/hr. Patient had remained at baseline, and regular diet was started. By 24 hours, his serum sodium concentration had reached 118 mEq/L. Attempts to relower the sodium concentration were made by starting 5% dextrose in water intravenously at 200 mL/hour. Sodium concentration fluctuated in the range of 114-119 mEq/L over the next 48 hours. Serum sodium concentrations gradually increased by 3-4 mEq/L per day to 123-128 mEq/L from day 4-7 before normal values were noted by day 12.

**Figure 1:**
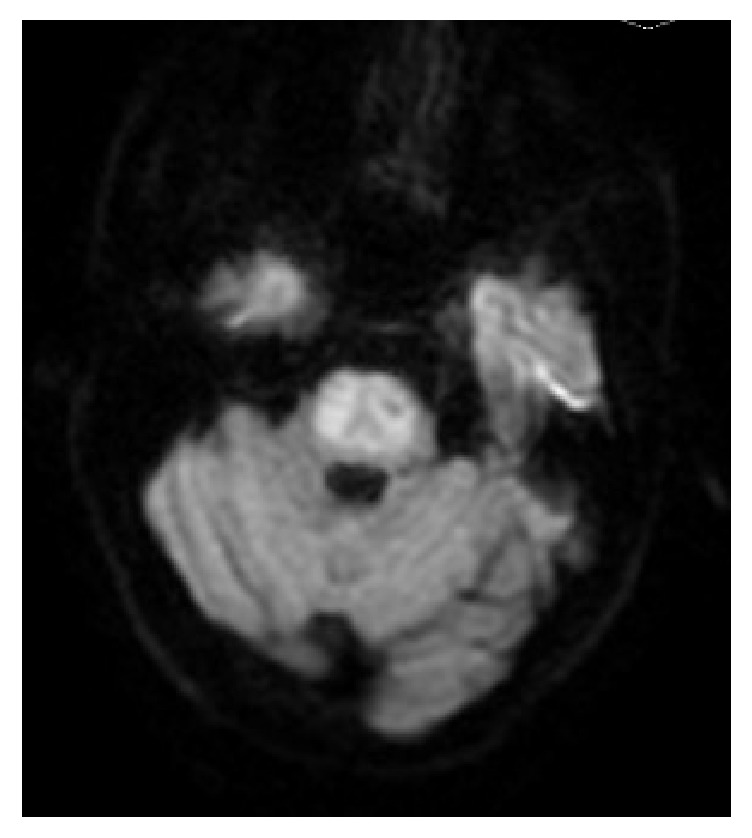
Magnetic Resonance Imaging of the brain without contrast showed high T2 signal and low T1 signal with restricted diffusion along with sparing of the bilateral peripheral pons, classic for central intrapontine, and extrapontine myelinolysis.

**Figure 2:**
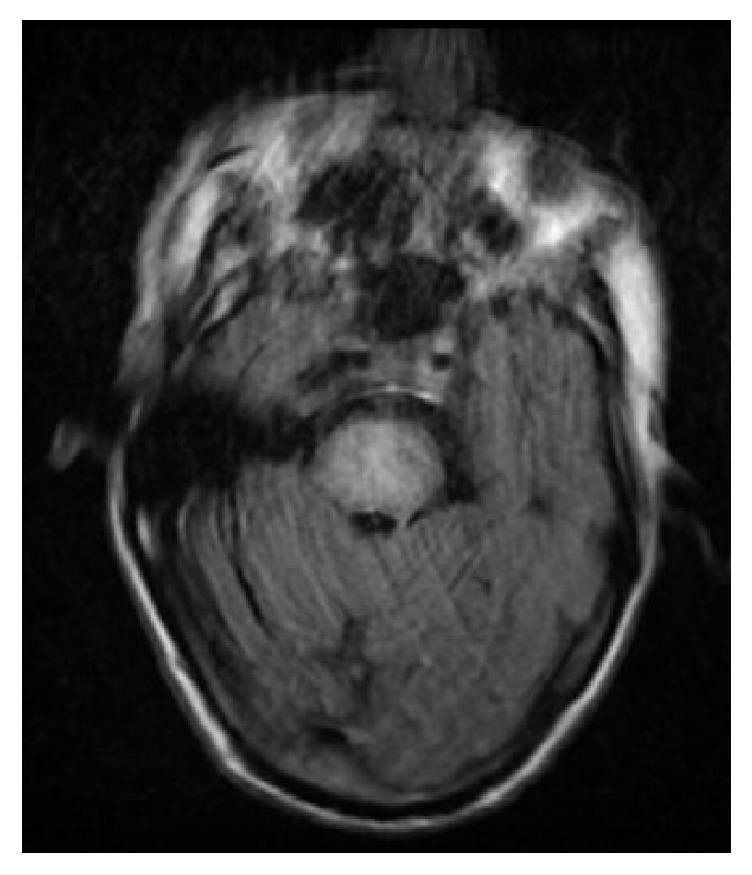
Magnetic Resonance Imaging of the brain without contrast showed high T2 signal and low T1 signal with restricted diffusion along with sparing of the bilateral peripheral pons, classic for central intrapontine, and extrapontine myelinolysis.

During the initial 6 days of the presentation, patient remained awake, alert, oriented to person and place. However, he then became somnolent and uncooperative with physical examination, experienced urinary incontinence, and ultimately developed changes in speech, increased tone of the upper extremities, and paraplegia by day 13 of the presentation. During this time, he also failed swallow evaluation requiring nasogastric tube (NGT) placement. CT of the brain without contrast was repeated which did not show any acute pathology. MRI of the brain could not be performed initially due to technical reasons. Over the ensuing days, he lost the ability to vocalize any words, could only answer by nodding, and was unable to elevate his upper extremities initially before losing the ability in the lower extremities. MRI of the brain performed on the third week of presentation revealed changes pathognomonic for central pontine myelinolysis sparing the periphery of the pons confirming clinical suspicion [Figures 1-2].

Over the ensuing weeks to months, aggressive physical therapy, speech therapy, and nutritional support were continued on general neurological ward. One month from symptoms onset, he gradually started to show signs of improvement and was initially only able to form single words, then phrases, and then sentences. By one and a half months, except for dysphagia for which he relied on NGT feedings, he displayed marked improvement in tetraparesis. Modified barium swallow revealed silent aspiration with liquids with no reflexive response to aspiration events, requiring placement of Percutaneous Endoscopic Gastric (PEG) tube 2 months from presentation. He failed repeated swallow evaluations and the PEG tube remained in place for a period of 80 days until he was finally able to tolerate oral pureed diet.

## 3. Discussion

ODS is a rare neurological condition described by Adams et al. in 1950s. Most commonly ODS is seen following rapid overcorrection of serum sodium in patients with severe chronic hyponatremia (serum sodium <120 mEq/L). The majority of ODS cases occur with initial serum sodium concentrations of <105 mEq/L. Alcoholism, malnutrition, liver disease, hypoxia, and hypokalemia predispose these patients in developing ODS. In our patient, chronic alcoholism and malnutrition were likely contributing factors.

Hyponatremia is defined by the relative excess of water to the serum sodium concentration. Hypotonic hyponatremia affects the brain by causing entry of water into the brain resulting in cerebral edema [3]. However, cellular adaptation by the brain restores the brain volume by initially causing loss of electrolytes within a few hours (“rapid adaptation”) and then ultimately normalizing brain volume through the loss of organic osmolytes over several days (“slow adaptation”) [3]. In the case of chronic hyponatremia, rapid correction of the serum sodium leads to increased extracellular tonicity, fluid shift to the extracellular compartment, and dehydration of the brain cells due to little adjustment time [3, 17]. Since oligodendrocyte is more susceptible to this type of damage, it leads to degeneration and destruction of the myelin [14].

The clinical manifestations of ODS are typically delayed for 2-6 days following the insult. As with our patient, initial symptoms may present with depressed level of awareness, dysarthria or mutism, and additional symptoms onset over the ensuing 1-2 weeks including quadriparesis, impaired sensation, and difficulty with coordination. At its most severe state, myelinolysis can lead to coma, “locked-in” syndrome, and death.

MRI is often the imaging of choice. In our patient, T2-weighted MRI revealed classic hyperintense or areas of demyelination of the pons consistent with ODS. Unfortunately, we initially could not perform the MRI until third week of symptoms onset due to technical reasons. However, our suspicion remained high and we continued with aggressive supportive measures in view of the symptoms. Delayed imaging is preferred to confirm the diagnosis since conventional imaging (MRI and CT) lag clinical manifestations in the first two weeks [10].

Our patient's unique presentation makes this case noteworthy with respect to detailed timing of the events. Following the initial pathognomonic symptoms described, he gradually displayed improvement and ultimately, making a remarkable, full recovery of tetraparesis by one and a half months. Despite marked improvements in motor and speech, dysphagia persisted for almost four and a half months and he remained dependent on NGT feedings and later PEG tube feeds. However, repeated swallow evaluations were performed periodically despite multiple failed attempts. As Louis G et al. demonstrated in a cohort of 36 patients with ODS managed in critical care setting, 32 of the patients required mechanical ventilation, 69 percent survived, and 56 percent of the survivors were left with only minimal neurologic sequelae. Furthermore, the initial severity of the illness was not a predictor of long-term prognosis [21]. Singh et al. performed a thorough search of the literature using multiple databases to identify all case series of ODS patients published from 1959 to January 2013. 2602 articles were identified comprising of 541 patients with ODS of whom 51.9% had favorable recovery and death in 24.8% [2].

Therefore, regardless of the initial severity of symptoms, we urge for aggressive early management including daily physical therapy, speech therapy, and optimal nutritional support including early PEG tube placement as opposed to seeking early comfort measures. The incremental improvements in mentation and functional capacity take months to be noticed. We strongly suggest that similar patients' needs to be aggressively cared for since complete recovery are possible.

## Figures and Tables

**Table 1:** Serum sodium, potassium, chloride, and bicarbonate concentrations over time.

Time	Sodium (mEq/L)	Potassium (mEq/L)	Chloride (mEq/L)	Bicarbonate (mEq/L)
0 hour	102	2.4	54	38

8 hours	106	2.9	63	33

16 hours	112	2.4	71	33

24 hours	118	3.0	82	28

48 hours	119	3.8	86	24

12 days	141	4.1	107	20
